# Experiences of Iranian physicians regarding do not resuscitate: a directed-content analysis

**Published:** 2016-08-23

**Authors:** Mohammadali Cheraghi, Fatemeh Bahramnezhad, Neda Mehrdad

**Affiliations:** 1 Associate Professor, Faculty of Nursing & Midwifery, Tehran University of Medical Sciences, Tehran, Iran;; 2 PhD Candidate in Nursing, Faculty of Nursing & Midwifery, Tehran University of Medical Sciences, Tehran, Iran;; 3 Associate Professor, Endocrinology and Metabolism Research Institute, Tehran University of Medical Sciences, Tehran, Iran.

**Keywords:** *Physician*, *DNR*, *Religion*, *Ethics*, *Legal*, *Iran*, *Directed content analysis*

## Abstract

One of the major advances in medicine has been the use of cardiopulmonary resuscitation (CPR) procedure since the 1960s in order to save human lives. This procedure has so far saved thousands of lives. Although CPR has helped to save lives, in some cases, it prolongs the process of dying, suffering, and pain in patients.

This study was conducted to explain the experience of Iranian physicians regarding do not resuscitate order (DNR). This study was a directed qualitative content analysis which analyzed the perspective of 8 physicians on different aspects of DNR guidelines. Semi-structured, in-depth interview was used to collect data (35 to 60 minutes). First, literature review of 6 main categories, including clinical, patient and family, moral, legal, religious, and economic aspects, was carried out through content analysis. At the end of each session, interviews were transcribed verbatim. Then, the text was broken into the smallest meaningful unit (code) and the codes were classified into main categories.

The codes were classified into 6 main categories, which were extracted from the literature. In the clinical domain 4 codes, in patient and family 3 codes, in moral domain 4 codes, in religious domain 3 codes, and in economic domain 1 code were extracted.

According to the findings of this study, it can be said that Iranian physicians approve the DNR order as it provides dying patients with a dignified death. However, they do not issue DNR order due to the lack of legal and religious support. Nevertheless, if legislators and the Iranian jurisprudence pass a bill in this regard, physicians with the help of clinical guidelines can issue DNR order for dying patients who require it.

## Introduction

One of the major advances in medicine has been the use of cardiopulmonary resuscitation (CPR) procedure since the 1960s to save human lives. Thus far, this procedure has saved thousands of lives (1). Although CPR has helped to save lives, in some cases, it prolongs the process of dying, suffering, and pain for patients (2). Prolongation of the dying process, in addition to the pain and suffering, has caused fundamental challenges for the patients' families and the health care system (3). The challenges in this regard include transference of patients to the hospital, hospitalization in the intensive care unit (ICU), use of equipment and facilities, intensive care bed occupancy despite urgent need of these beds by other patients, experience of severe emotional trauma by the patients and their families, moral, legal, ethical, and religious involvement, and job burnout of health care providers for patients who have less than 5% chance of survival or in case of survival will have a low quality of life (QOL) (1, 4, 5). Prescription of antibiotics, intravenous feeding, mechanical ventilation, and other care measures, in addition to futile care, is associated with legal, moral, ethical, and emotional challenges for the patients, their families, the treatment team, and the community (3). The purpose of a do not resuscitate (DNR) order for dying patients, is in fact to provide conditions for a comfortable death and avoid futile care measures (6). The four key ethical principles of autonomy, beneficence, justice, and non-malfeasance are important in the care of all patients. Moreover, they are in compliance with the clinical ethics of the Josephson Forum (principles of honesty, integrity, keeping promises, fairness, compassion, respect and reverence for others, responsible citizenship, justice, accountability, and responsibility) (7). The usefulness principle shows that health care providers should consider the benefits and advantages in providing care.Failure to achieve this goal will result in conflict with the principle of beneficence (8). Moreover, since the task of the treatment team is respecting patient autonomy and putting an end to patients’ pain and suffering, the principle of non-malfeasance appears to be very important. A dying patient is weak and vulnerable, and it is important that the treatment team is committed to not harming the patient during care delivery. Continuing treatments that prolong life, have not been requested by the patient, and likely are not benefiting him/her, and could harm the patient; therefore, they should be discontinued (9). Despite numerous discussions in this regard in many countries, the DNR issue is not completely resolved and is assigned to the physicians (as the main authorities). Physicians have moral and legal obligation to explain the patient's condition to him/her and his/her family (10). They should explain the prognosis of the disease and treatments which are futile and do not benefit the patient. Physicians have a duty to talk to the patient and his/her family about imminent death or intolerable disability, benefits, and medical expenses. With the consent of the patient and his/her family to start palliative care, the physician should clearly discuss interventions that prolong life and are not beneficial to the patient (11).

Insufficient education in this field and religious and moral issues are reasons for opposing DNR. Some physicians avoid talking to the patient for the fear that such a discussion with the patient may lead to the patient’s disappointment and his/her refusal of further treatment (12). However, the most important reason for the refusal of the treatment team to perform or issue DNR order is the lack of guidelines and legal support (13). In this regard, physicians and nurses believe that having a clear guideline and legal support can play a significant role in guiding these individuals and preventing many of the challenges associated with this issue (14).

In Iran, few studies have been conducted regarding DNR order. The authors did not find any qualitative study, which had explored this concept from different perspectives.

Decision about DNR in dying patients is a fundamental skill and includes many ethical, legal, and religious challenges. Perhaps, by determining the perspectives of health care providers, especially physicians, who are responsible for making the final decision in this regard, the development of a guideline suited to the Islamic society of Iran will be possible. Therefore, this study has been conducted to determine the perspectives of Iranian physicians regarding DNR for dying patients.

## Method

This study is part of a dissertation for a PhD degree in Nursing and Midwifery approved by Tehran University of Medical Sciences, Iran. The dissertation was developed in two phases. The first phase was literature review of the development of clinical DNR guidelines in dying patients. What is expressed in this paper is part of the second phase of the study (interviews with competent authorities regarding the guideline) and it is a directed content analysis. This paper has analyzed perspective of 8 Iranian physicians on different aspects of the formulation of DNR guidelines through individual semi-structured in-depth interviews. At first, based on content analysis of literature review on 6 main categories, including clinical, patient and family, moral, legal, religious, and economic aspects, guiding questions were prepared for each point of view. It should be noted that, after each question, aspects were deeply studied with the help of exploratory questions (Table 1). 

In this study, data collection was conducted through guided or theory-based content analysis described by Hsieh and Shannon (15), after collecting the data, the directed content analysis was used. In the first phase, studies and guidelines about DNR and particularly the Islamic and legal principles related to decisions about DNR were reviewed without time limitation.

**Table 1:** Interview questions based on the categories extracted from the literature review

**Categories**	**Questions**
**Clinical view**	What patient do you call a dying patient? What are the medical symptoms of death? Have you ever ordered DNR for your patients? What are the elements involved in CPR or DNR of dying patients?
**Patients’ and their families’ views**	Does the family have permission to decide on CPR or DNR of their patient? Is the family or patient permitted to question such an order? If a family disagrees with this order, what is the decision? If in the present context a family has such a request from the medical staff, how should they react?
**Moral view**	Based on the four ethical principles, what is the role of the patient and his/her family in this decision? Should they be satisfied? Is it necessary to consult with them?
**Legal view**	Does the DNR have any legal position in Iran? Can the legal guardian decide on this issue in Iran? If a patient has such a request, does the medical staff have immunity in case of implementation of such an order? Can such a decision be made based on a patient's will?
**Economic view**	Do economic elements play any role in CPR or DNR of patients?
**Religious view**	From the religion point of view, what are the limitations and boundaries of DNR order? Does Islam allow such an order?

*CPR= Cardiopulmonary resuscitation *

After reviewing the literature, six categories were extracted that were the basis for setting the interview guide and output codes resulted from the analysis were placed within the categories. Each interview lasted between 35 and 60 minutes, and data were collected during February-April 2015. At the beginning of each interview, the purpose of the study and the individuals’ right to refuse to participate in the study at any time during the interview were explained to the participants. In addition, oral informed consents were obtained from the participants and they were assured of confidentiality of data. At this stage, one of the participants rejected the recording of his interview by the researcher, so his interview was conducted by taking notes.

The transcription of interviews was implemented at the end of each session, and the transcripts were read several times in order to achieve a correct understanding. Then, the text was broken into the smallest meaningful units (codes) and the codes were classified into six main categories, which were extracted from the literature.

To ensure the credibility and acceptability of the data, the continuous involvement method with the research subject was used. In addition, the participants’ confirmation was used to verify the codes; the initial codes were checked by the interviewees before categorization. To provide the coding ability in the categories, peer check was used (by the advisor, consultant professors, and two PhD students).

## Results

In this study, 8 Iranian physicians (4 intensivists, 2 oncologists, 1 internist, and 1 neurologist) with the mean age of 46.12 ± 8.21 participated. 

A total of 300 codes were extracted from the interviews and, for a conceptual approximation to categories, were summarized in 15 codes and were placed in 6 categories. Futile care, burnout, fear of the law, lack of a single protocol and, protection of human dignity were the codes extracted in content analysis of texts, but other codes were also extracted during interviews with Iranian nurses.

**Clinical view**

The clinical view consists of independence in responding, attention to evidence-based arguments, ability to communicate with clients, and observing morality that can be achieved following individual-working environment interactions and interpersonal communication.

The following 4 codes appeared in this category.

*A) Burnout*

Burnout is associated with lack of energy and vitality, and leads to depersonalization, lack of personal status and individual progress, and poor job performance. This is an emotional, physical, and mental syndrome associated with a sense of low self-esteem. 

*[I have been embarrassed by being a physician again and again, because when you cannot do anything for your patient and see his/her suffering, you feel guilty. Every time, I say to myself, this time I will quit my job and go into business. I have lots of stress and a guilty conscience, so I feel depressed and absurd] *(Participant No. 3).

*B) Stereotyped care*

Stereotyped care means repeating care without combining theoretical and technical skills. 

Most participants believed that, today, it is impossible to offer the same treatment and cares for all based on books and articles and that treatment must be tailored to individual circumstances.

*[We do not have the critical thinking skills in dealing with patients, we have studied a series of articles and think that we should act based on them step by step. We never think. For example, in resuscitation, we act in such a way as if the one who invented resuscitation has prescribed it for all. The person who was the inventor of resuscitation, had tattooed No DNR on his chest]* (Participant No. 1).

*C) Futile care*

Effort and care with unattainable goals in which a degree of success is impossible, both in physiological and qualitative terms, and success or survival of patients is unknown is called futile care.

[*I have already performed more than hundreds of CPRs that were only for show and had no results. We have to do useless CPRs, of course. In the health system of our country, many measures are useless, including many CPRs. Even in the best hospital settings with the best staff and facilities, the success rate of resuscitation is roughly 10% to 15%, but here we resuscitate all patients]* (Participant No. 6).

*D) Lack of universal protocol*

Protocols aim to improve health care quality by reducing the costs and variety of care. Moreover, in the case of a variety of clinical evidence, they assist the healthcare team in the delivery of appropriate and effective care, and generate a clear understanding of decision-making models for competent authorities.

All participants in this study believed that, since there is no certain protocol in this regard, every physician acts arbitrarily.

[*In the field of diabetes or brain death, we have certain protocols and fixed procedures, and they really work, but not for DNR, so everyone does whatever he/she wants. Of course healthcare workers are careful not to fall into a trap. For example, some choose Slow Code, some do CPR for 45 minutes, some do not resuscitate at all, and just write that CPR was performed]* (Participant No. 2).

**Patients’ and their families’ views**

The patient and his/her family are the most important elements in decisions on DNR order, and without their permission, decision-making in this regard is hampered. This approach consists of 3 categories.

*A) Freedom of guilty conscience*

Guilty conscience is a sense associated with anxiety that is created in the unconscious mind of a person concerning the action he/she could have taken. Accordingly, the participants believed that the families resuscitate their patients until the last moment to escape from a guilty conscience.

*[I had a 75-year-old diabetic patient who had an accident. His GCS was 3 and he was hospitalized for 2 months, during which we had to resort to dialysis. I spoke with his son and explained that he has no way back, and if he comes back, he will die in the next few days, and his QOL will be zero. He was crying so bitterly and said I know that it is useless. So far, I have borrowed 10 million Tomans (Irans currency), but I am afraid that I will feel guilty tomorrow, and I am afraid my children will do the same with me in the future]* (Participant No. 2).

*B) Preservation of human dignity*

Human dignity is a sense of respect, value, and physical and mental integrity of individuals, and is considered one of the basic principles of patient rights. In this regard, the participants believed that one of the reasons that families agree with DNR order is to preserve the dignity of their patients. 

*[Often, families ask us to end their patient’s life by any means possible. They say we do not want our patient to die in such misery. For example, one patient was an important person and had dignity, he was a university professor; It was not permissible or fair to him that his human dignity be questioned]* (Participant No. 5).

*C) Lack of awareness of the family*

In fact, the inability to understand and experience events, thoughts and emotions evokes cognitive reaction conditions and accidents***. ***Participants in the study stated that families are not well informed about the disease process and prognosis; therefore, they try to save their patient until the last moment.

*[I had a patient with breast cancer who was not well at all. She was admitted to the ICU and her family was not allowed to visit her. Every time they asked personnel about the condition of their patient, personnel said trust in God, she will get well if God wills it. The family had sold their entire asset with the hope of recovery of their patient. When the patient died, her family was astonished. Her husband repeatedly asked: Why did you not tell me that my patient cannot survive? He said: If I had known, I would not allow her, myself, and my children to suffer so much]* (Participant No. 7).

**Moral view**

In medicine, morality is a set of ethical principles that affect the values and clinical judgment of physicians. These principles include respect for autonomy, beneficence, justice, and non-maleficence.

In the moral view, 2 codes of protecting the independence of patients and reliance on religious beliefs appeared.

*A) Protecting the independence of the patient*

Every human being has the right to decide freely without being under pressure, and with sufficient information about the measures that need to be taken. Most participants emphasized the independence of patients in decision-making and considered it as the most important moral principle.

*[One of the most important ethical principles in medicine is respect for autonomy of the patients. The patient should give the permission. I think that healthy individuals should decide what they want to happen after their death. For instance, his/her wish should be recorded in a form like organ donation, on a card or bracelet, or as a text on his/her driving license. You cannot decide about the asset of another person]* (Participant No. 3).

*B) Reliance on beliefs*

Morality is one of the main elements of human identity. The beliefs of the general population, social contract, and religious beliefs have led to the formation of morality. 

*[The Iranian people are emotional and have always struggled to keep their patients alive by every possible means. The opposite is also true. If they were asked to sacrifice and it meant the death of their beloved patient, they would do it.]* (Participant No. 6).

**Legal view**

Law is a set of instructions that is implemented by a set of institutions, acts as a mediator and facilitator of social bonds between people, and regulates human behavior. All participants in this study considered lack of legal support as the most important principle of non-compliance with DNR.

*[Why should we get ourselves in trouble? When the Medical Council and forensic medicine do not support us, why should we seek trouble? You may provide thousands of scientific arguments and even moral arguments for not resuscitating the patient, but the law will not let you go. You cannot satisfy them. Organ donation has been accepted only because of legal support and nothing else]* (Participant No. 8).

**Religious views**

Religion is a set of ideas, rules, and regulations that covers all insight-related, trend-related, and ethics-related principles of humanity. The following 4 codes were extracted from this view.

*A) Religion equal to logic*

Religion consists of ways, methods, and principles that contribute to the understanding of the issues of faith, and explains religious principles. However, logic is the knowledge by which to identify and present the right way of thinking and reasoning. In fact, in this code, participants believed that faith and reason both try to show individuals the right path.

[*Divine religions are exactly equal to wisdom and reason. When reason says a patient will not survive and his/her survival will cause damage, religion says the same. Religion is not opposed to logic. For example, euthanasia is logically hated and objected, and Islam and other main religions have strictly forbidden it. Thus, religion and logic are homogeneous]* (Participant No. 2).

*B) Miracle*

Miracle, in the public’s view, means surprising, unusual, and supernatural events. In divine religions, it is an extraordinary and supernatural event that occurs by the power of God or his envoys, and cannot be explained through natural rules of science.

*[I am not a religious person, but I believe the universe can do anything. The sound of a little baby may force the universe to raise his/her end stage mother. This cannot be denied. About 10-15 years ago, I saw a car fall off a cliff and all passengers die, except a 20 days old infant without even the smallest scratch. When we see these things, we feel doubtful about DNR]* (Participant No. 5).

*C) Respect for the dying persons*

In the Islamic perspective, when a person is about to die, his/her relatives have duties.

*[In fact, in religious terms, it is very rewarding. If we claim to be Muslims, we should act like Muslims. I have passed courses in the seminary for 2 to 3 years. There are lots of traditions and recommendations about dying persons. Whenever I perform CPR for a dying patient, I remember them. I swear these useless CPRs are not only non-rewarding, but a sin. We must respect dying individuals]* (Participant No. 3).

**Economic view**

Hospital costs, including medical and accommodation costs, is one of the factors affecting the process of treatment, and its role is very clear. 

*A) Lack of appropriate allocation of resources*

*[Sometimes the patient’s family does not continue the treatment, because they cannot pay the treatment costs. Often, my colleagues do not give rare drugs to patients who clearly will not survive only for high expenses, whether the costs are covered by the family or the government. High expenses must be prevented, we must evaluate what and where to invest, and something the Iranian people are unable to do]* (Participant No. 6).

## Discussion

Participants’ interviews were coded and placed into the categories based on the categories extracted from content analysis of the literature review. 

In the clinical view, the 4 codes of futile care, burnout, protection of human dignity, and lack of single protocol were extracted. 

From the perspective of the participants of this study, resuscitation of patients who may not survive in qualitative and psychological terms may lead to staff burnout. In this regard, Embriaco et al. estimated burnout in physicians in ICUs to be between 20% and 60% .The main causes of burnout in these physicians were workload, frequent communication with colleagues and patients’ families, and futile care (16). Hamric and Blackhall found that the main reason for burnout in physicians is moral challenges in the ICU atmosphere (17) .Physicians participating in the study believed that sometimes their efforts are not in line with their clinical commitments, have no benefit for their patients, and are considered futile (17). In this regard, Wilkinson and Savulescu believe that, although the decision to discontinue or not to start a medical treatment has excessive complexity and high load of value, physicians are morally responsible to stop treatments that are futile or not beneficial enough for patients (18).

The stereotyped care code was consistent with the findings by Brindley (3). This study emphasizes on performing CPR individually. CPR should be performed based on the patient's condition, the medical team agreement, and consent of the patient and his/her family, not based on theoretical findings in books and articles (3). O’Neill et al. stated that care services have inferior quality in nursing houses and are not performed according to the patient's needs and desires, but are repetitive and routine (19). In this regard, Papes et al. believe that routine intervention is a serious defect in nursing (20). They believe it should be replaced by patient-oriented care with an emphasis on maintaining the patient’s integrity and unique needs, along with the provision of specialized nursing process (20). 

The researcher did not find any studies on stereotyped care in medical treatment.

Huang et al. performed a study on a protocol for DNR in dying patients (21). Their findings showed that patients with DNR order have lower rates of oxygen consumption, inotropic drugs, chest X-rays, dialysis, and antibiotics consumption, and the order reduces unnecessary and futile care in these patients (21).

Song et al. in a study on decisive factors in DNR among cancer patients in Korean hospitals, found a significant relationship between the level of awareness in the family and acceptance of DNR (*P* <0.05) (22). Downar et al., after 44 semi-structured interviews with dying patients, stated that patients with higher level of awareness are more likely to accept DNR (23).

Physicians who participated in the study believed that families accept DNR order to preserve the human dignity of their patients, and accept the order if they are recommended to do so. Lee et al. performed a study on the protection of human dignity entitled "awareness and ethical attitudes toward DNR for cancer patients" (24). They concluded that 41.1% of cancer patients and their families accept DNR to maintain human dignity and ensure a dignified death in their patient (24). Mularski et al. performed a study on the quality of death in the ICU from the perspective of family members (25). They argued that the main reason for acceptance of the DNR order by family members was to ensure a dignified and peaceful death for the patients and protect their human dignity (25).

On the code of freedom of guilty conscience in family members, the researchers did not find any studies. However, Kelly believes that physicians and nurses feel guilty when ordering the slow code in cases where resuscitation is useless for patients (26).

In medical procedures, different wishes and opinions are involved in decision making; thus, each one may have a level of autonomy. By the early twentieth century, medical practice was based on a sort of patriarchy. This means that physicians had the right to act as a father based on the medical condition of the patient, and perform treatments even if they were not accepted by the patients. However, today, the patient has autonomy, independence, freedom, and respect, which are considered the principles of medical ethics (27). 

The finding of this study showed that physicians pay special attention to the principle of patient autonomy. Nevertheless, Pellegrino (28) and Rachels (29) argued that in western countries, respecting the principle of independence is largely accepted, whereas in other cultures, including that of Middle Eastern countries, sometimes the physician or the patient's relatives limit the patients’ independence. If DNR is requested by a patient, in order to respect the principle of patient independence, this must be accepted by the medical staff (30).

Curtis and Burt conducted a study on informed consent and the DNR order (31). They found that some physicians prefer to implement DNR order without the informed consent of the patients due to lack of legal support. In many countries, even obtaining an informed consent from the patient is not considered a legal support for the physician (31).

Some physicians may insist on life prolongation measures for religious reasons. However, this is contrary to the principle of morality, and the physician must listen to the patient’s request, even though he/she may oppose it for religious reasons (32). 

Also Frost et al. believes that one of the main reasons that may prevent patients from accepting DNR is religious beliefs. People who have strong religious beliefs are less willing to accept DNR (33). 

According to Pentz et al., the main reason for starting the debate on DNR is physicians’ beliefs (34).

From the economic view, participants believed that millions of national capital is annually spent on useless resuscitations.

Karnik et al. believe that the influence of economic factors on decision-making about DNR is inappropriate, and financial problems should not have a role in decision-making in this issue (35). 

In the health system, resources are sometimes allocated based on patients’ demands, rather than their real needs, while the allocation of resources in the health system must cover the real needs of individuals. Especially in recent years, in which the health reform plan has been implemented, the researcher believes that the fair and equitable allocation and distribution of health facilities is very important. Therefore, planning for the use of health resources for preventive measures may be necessary.

Lack of access to competent individuals who had completed specialized courses was one of the limitations of the research. However, the researchers tried to study experts who met the criteria (clinical specialists in this field who had scientific publications on the subject). Nevertheless, researchers often did not have access to these specialists. In addition, since this issue has moral challenges, the participants did not wish to speak in this regard in the early stages and preferred not to talk about their personal experiences. However, the researchers tried to solve this problem by assuring them of the confidentiality of the information.

## Conclusion

According to the results of the present study, it seems that physicians believed resuscitating some patients is useless and often resuscitating a patient who has no chance of survival compromises his/her dignity and creates many challenges for healthcare system. It seems that developing a clinical guideline with legal support can resolve many problems of the DNR order. In fact, developing an Islamic-Iranian guideline can prevent the implementation of futile and useless care.

It is suggested that more research be conducted on this subject to determine the perspectives of other health care providers as well as lawyers, clerics, patients and their families in order to make an accurate decision about DNR order.
